# Effects of grandparents’ involvement on young children’s resilience: mother’s parenting stress and family strength as mediators

**DOI:** 10.1186/s41155-025-00370-1

**Published:** 2025-11-19

**Authors:** Jinjing Wu, Junyi Zhu, Yan Lei, Leishan Shi

**Affiliations:** 1 Faculty of Teacher Education, Lishui University, No. 1, Xueyuan Road, Lishui City, 323000 Zhejiang Province PR China; 2 Faculty of Music, Lishui University, No. 1, Xueyuan Road, Lishui City, 323000 Zhejiang Province PR China

**Keywords:** Grandparents’ involvement, Resilience, Mother’s parenting stress, Family strength, Young children

## Abstract

**Background:**

Given the fact that grandparents share the responsibility for child-rearing in contemporary China, limited research has explored the relationship between grandparents’ involvement and grandchildren’s resilience in the three-generation family (a child lives with parents and one or more grandparents). The purpose of this study was to examine the relationship between grandparents’ involvement and young children’s resilience, as well as the mediating roles of mother’s parenting stress and family strength in this association.

**Methods:**

The study surveyed 919 mothers of children aged 3–5 years in Chinese three-generation families to report their children’s resilience, grandparents’ involvement, mother’s parenting stress, and family strengths.

**Results:**

The findings revealed a positive association between grandparents’ involvement and young children’s resilience. This relationship was partially mediated by mother’s parenting stress and family strengths independently, as well as sequentially through the pathway from mother’s parenting stress to family strengths.

**Conclusions:**

Grandparents’ involvement may help to relieve perceived mother’s parenting stress and enhance family strengths, thereby, indirectly affect young children’s resilience. These findings highlight the potential positive impact of grandparents’ involvement on young children’s resilience and offer a novel perspective for family counseling.

Viewed through the lens of positive psychology, Resilience emphasizes the exploration of an individual’s intrinsic potential, focusing on their capacity for self-transcendence and adaptive growth in the face of stress and adversity. Resilience is characterized by a set of protective mechanisms that enable successful adaptation in the face of adversity (Ernst & Burcak, 2019), yielding positive outcomes in child development, including enhanced individual resilience (Lockwood et al., 2015), improved well-being, stronger attachment (Karreman & Vingerhoets, 2012), and reduced depression and anxiety (Ma et al., 2020). Although resilience can be cultivated at any age, specific developmental windows offer optimal opportunities for fostering protective factors and leveraging protective systems to adapt effectively (Masten, 2001). Early childhood, marked by surging brain plasticity (Southwick & Charney, 2012), stands out as one such critical period. Furthermore, the resilience developed during early childhood significantly influences adaptation throughout childhood, adolescence, and even into adulthood (Werner, 1989).

Resilience in young children includes their dynamic responsiveness to environmental stressors, manifesting in the ability to adapt flexibly to maintain or enhance equilibrium by regulating self-control (Naglieri et al., 2013), and includes such aspects as initiative, self-regulation, and attachments/relationships. While researchers have focused on young children’s resilience, it has been primarily explored within the context of intergenerational relationships between parents and young children.

In China, the caregiving burden for preschool-aged children has emerged as a primary constraint on women’s labor market participation (Zhao & Bi, 2025). Mothers, while balancing employment and household responsibilities, undertake the majority of childcare duties, which impacts the quality of child-rearing and heightens perceived parenting stress (Skreden et al., 2012). Concurrently, urbanization-driven shifts in intergenerational family dynamics have fostered a distinctive childcare support system, characterized by increasing grandparental involvement in grandchild care (Goh & Kuczynski, 2010; Shen, 2023). Data indicate that 69.3% of grandparents contribute to grandchild rearing (Han & Lyu, 2025), forming a collaborative intergenerational childcare model unique to China (Chen et al., 2011a, b). Child-rearing practices in contemporary Chinese families not only differ significantly from those of the past, requiring higher costs and more time, but also diverge markedly from those of Western societies characterized by nuclear family structures and individualism. The Chinese approach to “intensive motherhood” is built on the foundation of selfless “intensive grandmotherhood.” More precisely, it relies on an intergenerational relay of caregiving, predominantly provided by women from both paternal and grandpaternal lineages (Ji et al., 2020).

Mother’s Parenting Stress is generally associated with adverse effects on family quality, functioning, and relationships (Crnic & Greenberg, 1990). During early childhood, the family serves as a critical system intricately linked to child development, with the family environment widely recognized as a pivotal social determinant of children’s psychological development (Bronfenbrenner, 1979). Among family factors influencing early childhood development, family strength, a key psychological dimension of the family environment, is particularly salient. Enhancing parental support strengthens family strength, fostering children’s resilience in adversity (Westphaln et al., 2022). Higher family strength during early childhood is associated with greater resilience in young children (Lee & Moon, 2011; Kang & Lee, 2014). In diverse co-parenting arrangements, intergenerational co-parenting relationships exhibit notable complexity (Chen & Du, 2023). In grandparental co-parenting models, children form not only parent-child attachments but also stable emotional bonds with grandparents, establishing grandparent-grandchild attachment (Poehlmann, 2003). Research indicates that in families with robust co-parenting dynamics, grandparents’ involvement mitigates mother’s parenting stress and enhances family cohesion, thereby promoting child development (Luo et al., 2020). Supportive and nurturing grandparental caregiving styles further facilitate the development of young children’s peer interaction skills (Song et al., 2016). However, some scholars argue that grandparents’ involvement and co-parenting models may adversely affect grandparents’ health (He et al., 2021) and psychological well-being (Liu & Wang, 2022).

Despite the recognized significance of grandparents’ involvement in early childhood development, few studies have investigated the association between grandparents’ involvement and young children’s resilience.

## Grandparents’ involvement and young children’s resilience

Grandparents’ involvement is the extent to which grandparents are engaged and participate in the lives of their grandchildren (Dunifon et al., 2018). Grandparents play an active role in the upbringing and care of their grandchildren through their developmental support; routine care; and leisure-coaching, participatory (consumption-based), and cyber-parenting activities (Choi, 2016). Long shaped by Confucian principles, Chinese individuals prioritize familial obligations, and families reciprocate by providing care and support (Lou & Wang, 2016). Due to the shortcomings of China’s social service system for the elderly, familial support remains the primary solution for addressing old age-related challenges. Chinese grandparents often view caring for their grandchildren as a favorable option for an intergenerational exchange (Sun & Jiang, 2017). Consequently, grandparents with grandchildren frequently support their adult children in grandchild rearing (Leeson, 2018), with data indicating that 63% of grandparents in China actively participate in this role (Han & Lyu, 2025).

In accordance with ecosystem theory, grandparents serve as auxiliary parenting figures for young children, intervening in the mother–child dyadic system to establish a mother–child–grandparent ternary system. Along with the involvement of the young children’s mother and family, this constitutes the closest microsystem influencing young children’s development. Grandparents’ involvement centers on the interpersonal interactions among family members and children within the family unit, emphasizing the transfer of parenting across generations (Belsky, 1984). These interactions form the foundation for the connection between grandparent involvement and young children’s resilience. Grandparents’ tolerance, understanding, and thoughtfulness serve as exemplary parenting qualities, establishing a developmental triadic system (Bronfenbrenner, 1979). Research indicates that children often harbor positive feelings toward their grandparents as primary caregivers (Downie et al., 2010), and the favorable attachments they cultivate with their grandparents can directly enhance their social adjustment (Hayslip et al., 2019). Grandparents’ involvement fosters essential facets of young children’s resilience, including their cognitive skills, positive self-worth, and family cohesion (Egeland et al., 1993). However, these findings have primarily focused on school-aged children.

Although few studies have directly investigated the relationship between grandparents’ involvement and young children’s resilience, indirect evidence provides some support for this association. Studies indicate that such involvement positively influences young children’s self-control and emotional regulation (Chen & Jiang, 2019). Grandparents’ involvement is associated with enhanced initiative in toddlers (Lee et al., 2015; Rahmawati & Diana, 2016). These studies prove that grandparents’ involvement in early childhood parenting can positively influence young children’s self-resilience, affirming the notion that such involvement in parenting can benefit young children’s resilience.

### Mother’s parenting stress as mediator between grandparents’ involvement and young children’s resilience

Mother’s parenting stress is the stress mothers experience in fulfilling their maternal roles and during parent–child interactions. This stress is influenced by their personal characteristics, interactions with their children, their children’s traits, and situational factors within the family. It manifests in parenting difficulties, dysfunctional interactions, and the challenges presented by difficult children (Abidin, 1990). In China, mothers bear a greater share of the responsibility for parenting and household chores, leading to numerous challenges in balancing their maternal roles with their work and family responsibilities. Mothers not only endure the stress resulting from the continual accumulation of daily pressures in their lives (Daundasekara et al., 2021), but they must also contend with the negative psychological effects of their maternal role’s demands (Xiang et al., 2014). Working mothers in particular experience heightened parenting stress due to social support deficits, exacerbated by China’s fledgling parental leave system and inadequate social care services (Chen et al., 2011a, b; Yang et al., 2019).

According to ecosystem theory, the mother–toddler relationship is a crucial dichotomous system in early childhood development (Bronfenbrenner, 1979). The more parenting stress a mother experiences, the more she can reject her children (Lee, 2008) and adopt severe parenting attitudes, such as forced physical punishment or permissiveness (Lee & Mun, 2011). Clearly, mothers with high parenting stress experience negative emotions and engage in negative parenting behaviors (Abidin, 1990), affecting their children’s emotional and psychological well-being (Fadzil & Maulidiyah, 2023), which can then negatively impact the development of resilience in young children (Choi & Lee, 2011).

According to family systems theory, within the entire family system, the involvement of other adult caregivers besides the mother (such as the father, grandparents, or members of the extended family) expands the mother-child relationship into a triadic relationship. When these caregiving participants establish harmonious relationships, achieving systemic equilibrium, children derive developmental benefits (Minuchin, 1985). Interdependence theory further posits that interpersonal experiences are fundamentally shaped by interactions, with relational partners mutually influencing each other’s behaviors. As a result, individuals form perceptions tied to their experiences and the quality of their relationships (Rusbult & Buunk, 1993; Polenick et al., 2017). This theory provides critical insights into the reciprocal influences among parents’ and grandparents’ experiences, perceptions, and emotional closeness. By integrating family systems theory and interdependence theory, this study has developed a more comprehensive analytical framework. Grandparents’ involvement in parenting not only supports mothers in parenting more effectively, instills the family culture, and fosters strong bonds between the three generations, but also reduces parenting stress for mothers of young children (Kim et al., 2015). As counselors, grandparents can offer guidance on young mothers’ parenting attitudes. Grandparents can also support mothers of young children by alleviating their household chores through active involvement in their daily lives. Therefore, grandparents’ participation in parenting mitigates the psychological burden on young mothers (Noriega et al., 2017). Grandparents’ involvement in child rearing directly affects mother’s parenting self-efficacy, and mothers who raise young children themselves have higher self-efficacy (Wang et al., 2015). However, studies also indicate that mothers experience increased anxiety and parenting stress when grandparents are involved in co-parenting (Sivak, 2018; Wang et al., 2020). Research has examined the mediating role of mother’s parenting stress in the relationships between grandparents’ involvement and various young children outcomes, including problem behaviors (Li et al., 2016), personality and adaptation (Sun & Jiang, 2017), social adjustment (Luo et al., 2020), and social competence (Gong et al., 2021).

The impact of grandparents’ involvement in caregiving, mediated by mother’s parenting stress, on young children’s resilience remains inconclusive. Consequently, there is a pressing need to investigate the current state of grandparent-parent co-parenting in Chinese families, its influence on young children’s resilience, and the underlying mechanisms driving these effects.

### Family strength as mediator between grandparents’ involvement and young children’s resilience

Family strength refers to the inherent health and positive dynamics within families. According to ecosystem theory (Bronfenbrenner, 1979), the family environment involves the primary social context humans experience from birth, and is a significant factor in later development. Grandparents’ involvement is a crucial variable in the family environment during early childhood parenting. Such involvement involves frequent interactions between grandparents and their adult children, which strengthen family bonds. By imparting family culture to their grandchildren, grandparents foster a sense of cultural identity and family solidarity (Even-Zohar & Garby, 2016). Literature has observed that grandfathers in particular promote family identity and wholesomeness by addressing family members’ needs for strong, stable, continuous, disciplined, and communicative relationships, thus educating their grandchildren about family dynamics (Bates & Taylor, 2013a, b).

Psychological factors within the family environment are crucial predictors of young children’s future positive adjustment, and family strength is one of the most representative factors of these psychological components (Xu et al., 2007). A healthy family recognizes the individuality of its members, enabling clear, positive conversations; the effective resolution of family problems or crises; a clear delineation of responsibilities; and the establishment of shared family values and rules (Yoo et al., 2013). Therefore, families with healthy dynamics facilitate the better development of attachments/relationships in young children, promoting self-control as young children learn through home discipline (Chen & Jiang, 2019). Bonding within families, shared values and goals, positive communication, and problem-solving skills all significantly impact young children’s resilience (Dunifon et al., 2018). Research indicates that greater family strength correlates with higher resilience in young children (Lee & Moon, 2011).

However, the interplay between grandparents’ involvement, family strength, and young children’s resilience remains underexplored. Recent studies have explored the link between family strength and young children’s resilience (Yoon et al., 2018), as well as the impact of parental involvement behaviors on young children’s resilience (Kang & Kim, 2017). Hence, the current study hypothesizes that grandparents’ engagement behaviors contribute to the development of young children’s resilience by building family strength.

### Relationship between mother’s parenting stress and family strength.

Mothers’ parenting stress is an indicator of family health. An increase in the amount of stress that mothers experience in interactions with their children can negatively affect their sense of bonding, communication, and family cohesion and resilience. Specifically, the mother’s parenting stress can threaten family strength (Kim et al., 2007). Research indicates that mother’s parenting stress significantly and negatively impacts young children’s self-regulation and indirectly affects young children’s behavior through family strength (Yu & Choi, 2022). Mothers’ parenting stress influences young children’s social performance, mediated through family strength (Hong & Kim, 2020). Therefore, we can hypothesize that grandparents’ involvement may influence young children’s resilience through the interconnected relationship between mother’s parenting stress and family strength.

### Goal of the study

**Fig. 1:**
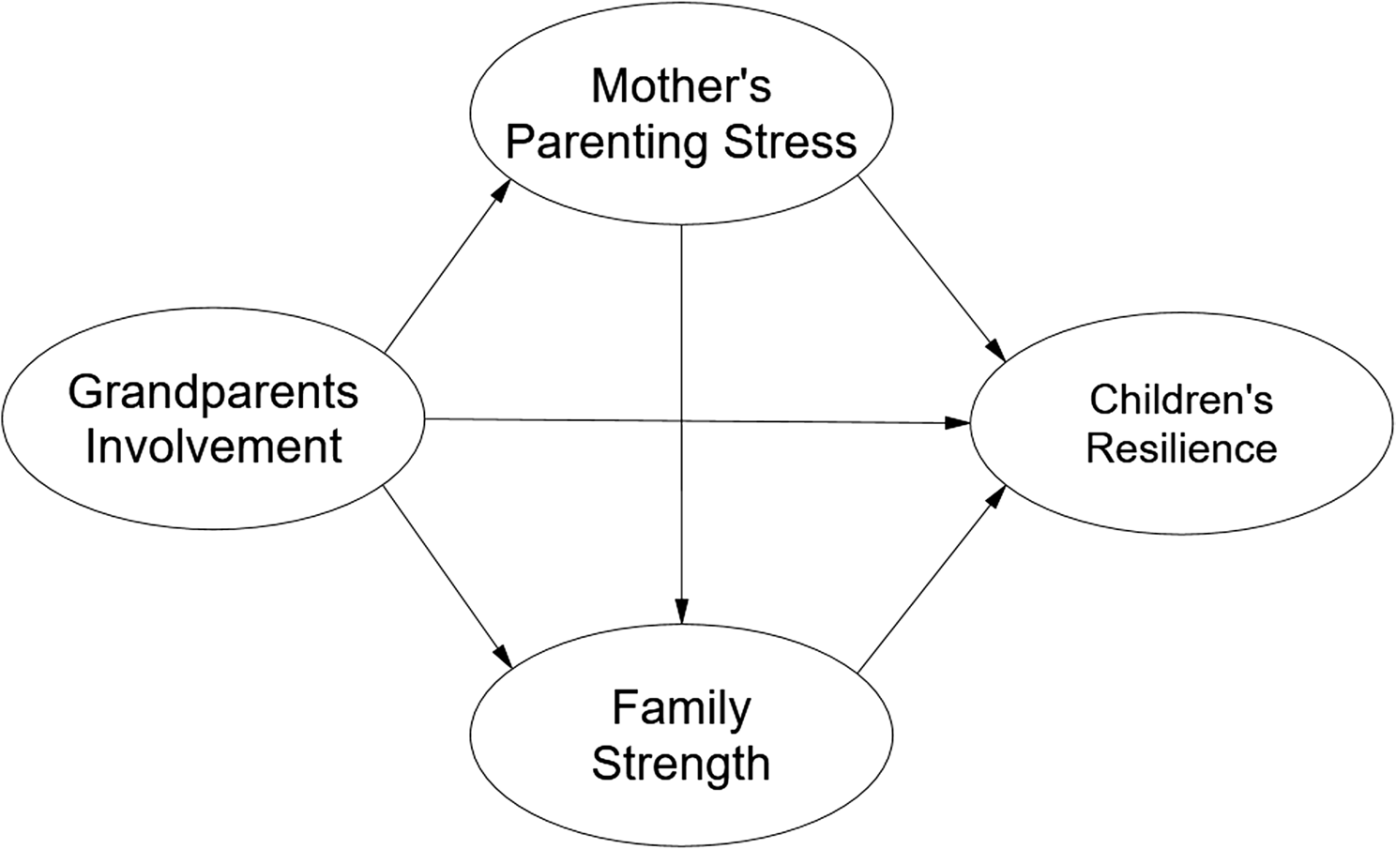
Theoretical research framework

Although research in ecological systems theory has demonstrated the positive impacts of grandparents’ involvement on young children’s psychological resilience, the relationship between grandparental involvement and young children’s resilience and its internal mechanisms remains underexplored. Previous studies have not simultaneously considered the relationships among variables at the grandparent, mother, family, and child levels, as well as the internal mechanisms by which grandparents’ involvement affects young children. Based on ecosystem theory, this study constructed a theoretical model to examine the effects of grandparents’ involvement on young children’s resilience, with mothers’ parenting stress and family strength as mediating variables (Fig. 1), to explore these effects and their underlying mechanisms. The study population consisted of young children aged 3 to 5 years. Control variables included the children’s age, gender (Chen et al., 2021), and birth order (Kour, 2022); the mother’s age, education, and occupation (Lee, 2018; Hong & Kim, 2020);and family type and monthly income (Bruno et al., 2023).

Accordingly, the following hypotheses are proposed:

- Hypothesis 1: Grandparents’ involvement positively relates to young children’s resilience.
- Hypothesis 2: Mother’s parenting stress mediates the relationship between grandparents’ involvement and young children’s resilience.
- Hypothesis 3: Family strength mediates the relationship between grandparents’ involvement and young children’s resilience.
- Hypothesis 4: Mother’s parenting stress negatively predicts family strength, and grandparents’ involvement also impacts young children’s resilience through the chain mediator of mother’s parenting stress and family strength.

## Research methodology

### Participants and procedures

Ethical approval for this study was obtained from the Ethics Committee of Lishui College. A stratified sample of 1,005 mothers in urban families with young children aged 3–5 years, whose grandparents were involved in parenting, was drawn from seven cities in Zhejiang Province: Hangzhou, Ningbo, Wenzhou, Jiaxing, Jinhua, Quzhou, and Lishui. The selection criteria were (a) the child resided with married parents and (b) at least one of the child’s grandparents was directly involved in the child’s parenting (Bowers & Myers, 1999; Dunifon et al., 2018). Parents who did not meet these two criteria did not participate in the survey. All participants were mothers of young children receiving support from their grandparents and were informed about the purpose of the study; as volunteers, they could withdraw from the study at any time without penalty. The questionnaires were completed by mothers using a web-based app, and the administration procedure was explained to them, assuring them of confidentiality and support for any issues. Based on completion time, 919 questionnaires were deemed valid, yielding a validity rate of 91.4%. Table 1 displays the participants’ basic information.

**Table 1:** Demographic information of the participants (*N* = 919)

Sample	Characteristics	Category	Number	*P* (%)
Children	Age (Years)	3	283	30.8
		4	308	33.5
		5	328	35.7
	Gender	Boy	473	51.5
		Girl	446	48.5
	Birth order	Only child	319	34.7
		First child	279	30.4
		Second child or above	321	34.9
Mother	Age (Years)	21–25	51	5.5
		26–30	147	16.0
		31–35	253	27.5
		36–40	283	30.8
		41–45	150	16.3
		46+	35	3.8
	Education	Middle school and below	61	6.6
		High school	71	7.7
		3-year college	259	28.2
		4-year university diploma	420	45.7
		Master or above	108	11.8
	Job	Full-time	695	75.6
		Freelance	165	18.0
		Part-time	24	2.6
		Unemployed	35	3.8
Grandparent	Type	Paternal grandfather	106	11.5
		Paternal grandmother	322	35.0
		Maternal grandfather	82	8.9
		Maternal grandmother	409	44.5
	Age (Years)	< 50	72	7.8
		51–60	362	39.4
		61–70	398	43.3
		71+	87	9.5
	Education	Elementary school or below	239	26.0
		Middle school	288	31.3
		High school	288	31.3
		3-year college or above	104	11.3
Family	Type	Nuclear family	569	61.9
		Stem family	263	28.6
		Other	87	9.5
	Monthly income (Yuan)	< 3,000	59	6.4
		3,000–6,000	123	13.4
		6,000–9,000	274	29.8
		9,000–12,000	344	37.4
		12,001+	119	12.9

### Research tools

#### Young children’s resilience scale

This study used the Second Edition of the Resilience in Young Children Scale developed by Naglieri et al. (2013); subsequently, Niu et al. (2019) adapted it for Chinese culture. The questionnaire consists of 27 questions categorized into three dimensions: initiative (YCR1) (e.g., “try different ways to solve a problem?”), self-regulation (YCR2) (e.g., “calm himself/herself down?”), and attachment (YCR3) (e.g., “show affection for familiar adults?”). A five-point Likert scale was used, with responses ranging from one (“does not do it at all”) to five (“does it very often”). The higher the score, the stronger the young children’s resilience. In this study, the resilience questionnaire’s Cronbach’s alpha was 0.943, and that for each dimension was 0.886, 0.915, and 0.917, respectively.

#### Grandparents’ involvement scale

This study also used the Questionnaire for Grandparent Involvement as developed by Choi (2016). The questionnaire consisted of 39 items related to five categories: development support activities (GI1) (e.g., “He/she explains to the children what they are curious about.”); daily care activities (GI2) (e.g., “He/she cares about how the child gets along with friends.”); leisure coaching (GI3) (e.g., “He/she goes with the child to the park, zoo, museum, etc.”); participatory (consumption) activities (GI4) (e.g., “He/she likes to think or talk with the child about something together.”); and cyber-parenting activities (GI5) (e.g., “He/she uses the Internet or media to find information about parenting.”). Items were also measured on a five-point Likert-type scale, with responses ranging from one (“strongly disagree”) to five (“strongly agree”). Although China and Korea share the same East Asian cultural background, this study examined the questionnaire in the Chinese cultural context. The questionnaire was developed by an expert panel and finalized following the cross-cultural adaptation process for self-report measures proposed by Beaton et al. (2000). The Cronbach’s alpha for the grandparent participation questionnaire in this study was found to be 0.957, with each dimension having Cronbach’s alpha values of 0.921, 0.894, 0.893, 0.879, and 0.859, respectively. The Average Variance Extracted (AVE) values were 0.762, 0.754, 0.638, 0.629, and 0.756, while the Composite Reliability (CR) values were 0.910, 0.816, 0.754, 0.652, and 0.858, respectively. Results of the confirmatory factor analysis (CFA) indicated acceptable model fit: CFI = 0.918, TLI = 0.902, RMSEA = 0.072 (90% CI = 0.066–0.083), and SRMR = 0.052.

#### Mother’s parenting stress scale

This study measured mothers’ parenting stress using the 15-item Short Form Parenting Stress Scale; Luo et al. (2021) developed this scale by adapting Abidin’s (1992) Parenting Stress Index Short Form for China’s national situation. The questionnaire includes items related to parenting distress (MPS1) (e.g., “Since having a child, I can hardly do what I like.”), dysfunctional parent-child interactions (MPS2) (e.g., “Most of the time I feel that my child does not like me and does not want to be close to me”), and dysfunctional parent-child interaction (MPS3) (e.g., “My child seems to cry more and be more fussy than other children.”). The items were also measured on a five-point Likert-type scale, with responses ranging from one (“never”) to five (“always”). In this study, the Cronbach’s alpha for the index was 0.939, with each dimension having Cronbach’s alpha values of 0.893, 0.926, and 0.887, respectively.

#### Family strength scale

This study then incorporated the Family Strength Questionnaire developed by Yoo et al. (2013). The questionnaire consists of 22 items, with five categories: family resilience (FS1) (e.g., “Families will help each other.”), valuing each other & acceptance (FS2) (e.g., “There is mutual respect and acceptance between families.”), qualitative bonds (FS3) (e.g., “Family members like to chat together.”), economic stability & cooperation (FS4) (e.g., “Our family has sufficient financial resources for leisure and cultural life.”), and caring about community (FS5) (e.g., “Our family has its traditions and culture.”). Items were also measured on a five-point Likert-type scale, with responses ranging from one (“strongly disagree”) to five (“strongly agree”). Similar to the Grandparent Involvement Scale, the Family Strength Questionnaire was tested for applicability to the Chinese cultural context following the same steps and requirements. The Cronbach’s alpha for this study’s Family Strength Questionnaire was 0.957, with each dimension having Cronbach’s alpha values of 0.934, 0925, 0.881, 0.913, and 0.873, respectively.

### Data analysis

The collected data were analyzed using SPSS software, version 26.0, and Mplus 8.3. The statistical test for significance was set as *p* < 0.05. The statistical analyses included descriptive statistics, a correlation analysis, structural equation modeling, mediating effect estimation, and bootstrapping. The model’s fit was assessed using such indicators as CFI, TLI, SRMR, and RMSEA (Byrne, 2001). A good fit was indicated if the CFI and TLI exceeded 0.90. The model’s fit was considered good if the RMSEA and SRMR were below 0.06, with values between 0.06 and 0.08 deemed acceptable (Byrne, 2001; Marsh et al., 2004). Bootstrapping with 1,000 samples was performed to assess the significance of the mediating effect and calculate the effect size (MacKinnon, 2009).

### Common method bias

The common method bias test using Harman’s one-way method yielded 16 factors greater than 1. The first factor explained 33.56% of the variance, which is below the critical criterion of 40% (Podsakoff et al., 2003), indicating no serious common method variation in the data.

## Results

### Correlations and descriptive statistics

Table 2 presents the variables’ descriptive statistics and correlation analyses’ results. Significant positive correlations were observed between young children’s resilience and both grandparent involvement and family strength, with correlation coefficients ranging from 0.073 to 0.689. Significant, negative correlations were identified between mothers’ parenting stress and each factor of young children’s resilience, grandparents’ involvement, and family strength, with correlation coefficients ranging from − 0.378 to 0.108.

**Table 2:** Correlation coefficients, means, and standard deviations of variables

	YCR1	YCR2	YCR3	GI1	GI2	GI3	GI4	GI5	MPS1	MPS2	MPS3	FS1	FS2	FS3	FS4	FS5
YCR1	1															
YCR2	0.606**	1														
YCR3	0.607**	0.462**	1													
GI1	0.295**	0.201**	0.226**	1												
GI2	0.251**	0.110**	0.109**	0.522**	1											
GI3	0.326**	0.147**	0.171**	0.544**	0.511**	1										
GI4	0.294**	0.152**	0.118**	0.553**	0.574**	0.682**	1									
GI5	0.275**	0.127**	0.073*	0.553**	0.470**	0.600**	0.689**	1								
MPS1	−0.252**	−0.200**	−0.117**	−0.166**	−0.235**	−0.159**	−0.137**	−0.201**	1							
MPS2	−0.378**	−0.375**	−0.325**	−0.250**	−0.266**	−0.225**	−0.187**	−0.147**	0.545**	1						
MPS3	−0.279**	−0.290**	−0.258**	−0.198**	−0.202**	−0.144**	−0.125**	−0.108**	0.600**	0.718**	1					
FS1	0.288**	0.252**	0.250**	0.215**	0.148**	0.219**	0.215**	0.182**	−0.058	−0.217**	−0.171**	1				
FS2	0.357**	0.372**	0.299**	0.201**	0.136**	0.226**	0.217**	0.203**	−0.140**	−0.252**	−0.177**	0.612**	1			
FS3	0.328**	0.353**	0.301**	0.165**	0.098**	0.193**	0.155**	0.134**	−0.047	−0.245**	−0.148**	0.582**	0.709**	1		
FS4	0.363**	0.360**	0.309**	0.096**	0.077*	0.119**	0.110**	0.077*	−0.092**	−0.197**	−0.135**	0.558**	0.680**	0.694**	1	
FS5	0.298**	0.280**	0.263**	0.133**	0.166**	0.201**	0.118**	0.123**	−0.147**	−0.264**	−0.164**	0.493**	0.536**	0.603**	0.587**	1
M	3.60	3.56	3.57	3.31	3.50	3.36	3.36	3.25	2.69	2.45	2.40	3.38	3.11	3.16	3.12	3.23
SD	0.73	0.77	0.83	0.85	0.89	0.87	0.81	0.90	0.86	0.99	0.93	1.04	1.12	1.08	1.19	1.09

*YCR1* initiative, *YCR2 *self-regulation, *YCR3 *attachment/relationship, *GI1 *development support activities, *GI2 *daily care activities, *GI3 *leisure coaching activities, *GI4 *participatory (consumption) activities, *GI5 *cyber-parenting activities, *MPS1 *parental distress, *MPS2 *dysfunctional parent–child interaction, *MPS3 *difficult child characteristics, *FS1 *family resilience, *FS2 *valuing each other and acceptance, *FS3 *qualitative bonds, *FS4 *economic stability and cooperation, *FS5 *caring about community

*p < 0.05, **p < 0.01, ***p < 0.001

### Hypothesis testing

#### Total effect

This study examined the effects of grandparents’ involvement on young children’s resilience by developing a structural equation model with good fit (χ2/df = 279.313/93 = 3.00, CFI = 0.944, TLI = 0.930, RMSEA = 0.0047 [90% CI 0.040–0.053], and SRMR = 0.056). After controlling for the subjects’ demographic variables, the results revealed that grandparents’ involvement significantly related to young children’s resilience (β = 0.372, *p* < 0.001, [95% CI 0.309, 0.436]), explaining 22.1% of the variance (*p* < 0.001).

#### Mediating effects

**Fig. 2:**
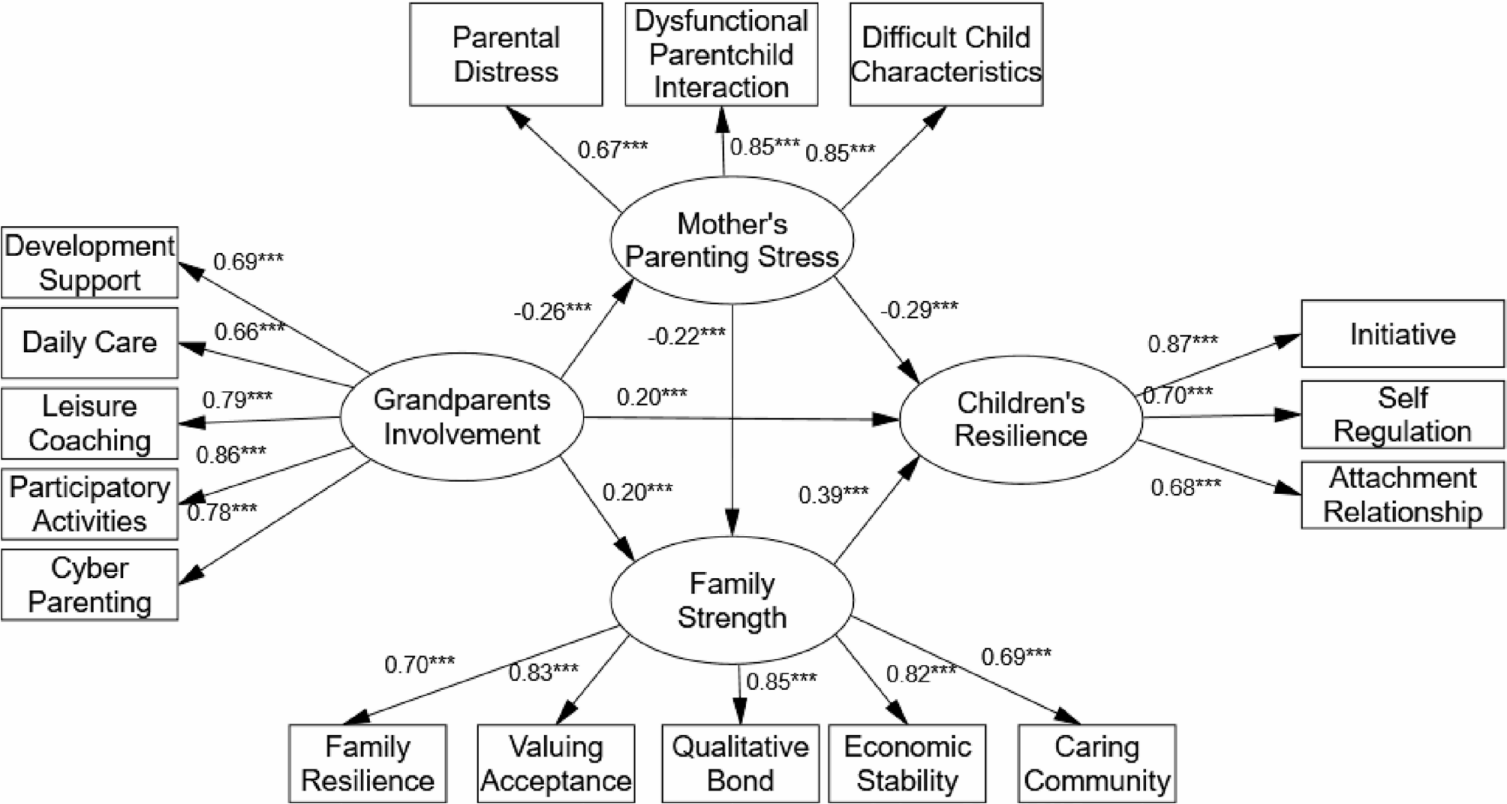
Results of the mediating effect

The mediating effect of mother’s parenting stress and family strength was further examined through the total-effects model using bias-corrected, nonparametric percentile bootstrapping (χ2/df = 835.55/255 = 3.28, CFI = 0.924, TLI = 0.911, RMSEA = 0.0050 [90% CI 0.073–0.093], and SRMR = 0.057). Figure 2 illustrates the coefficients of the effects for each pathway.

As Fig. 2 indicates, grandparents’ involvement negatively related to mother’s parenting stress (β = −0.261, *p* < 0.001, 95% CI [− 0.349, − 0.181]), positively related to family strength (β = 0.200, *p* < 0.001, 95% CI [0.126, 0.237]), and also positively related to young children’s resilience (β = 0.197, *p* < 0.001, 95% CI [0.114, 0.268]). Mother’s parenting stress negatively related to young children’s resilience (β = −0.293, *p* < 0.001, 95% CI [− 0.365, − 0.216], and family strength positively related to young children’s resilience (β = 0.385, *p* < 0.001, 95% CI [0.318, 0.459]). Mother’s parenting stress negatively related to family strength (β = −0.218, *p* < 0.001, 95% CI [− 0.295, − 0.135]).

The total effect of grandparents’ involvement on young children’s resilience was 0.372 (95% CI [0.294, 0.449]), with a total indirect effect of 0.175 (95% CI [0.134, 0.222]), which accounted for 47.1% of the total effect. Of the three pathways for the effect of grandparents’ involvement on young children’s resilience (Table 3), the M1, M2, and M3 pathways were all significant. The mediation effect was 0.077 (95% CI [0.049, 0.111]) for M1, 0.077 (95% CI [0.048, 0.108)] for M2, and 0.022 (95% CI [0.013, 0.034]). This suggests that mother’s parenting stress and family strength not only individually mediated the relationship between grandparents’ involvement and young children’s resilience, but also that the chained mediating effect of mother’s parenting stress and family strength was significant. Hence, Hypotheses 2, 3, and 4 are supported. However, testing the three mediating paths of grandparents’ involvement and young children’s resilience (M1, M2, and M3 in Table 3) revealed that the difference between the M1 and M2 paths was not significant (*p* = 0.98), but M1 > M3 (*p* = 0.001) and M2 > M3 (*p* = 0.000). This suggests that mother’s parenting stress and family strength individually mediate the relationship between grandparents’ involvement and young children’s resilience more strongly than the chained mediation of mother’s parenting stress and family strength.

**Table 3:** Mediated pathways and effect

Paths	Estimation	Bootstrapping 95% CI		Mediating effect proportion
		Lower	Upper	
M_1_: GI→MPS→YCR	0.077	0.049	0.111	20.7%
M_2_: GI→FS→YCR	0.077	0.048	0.108	20.7%
M_3_: GI→MPS→FS→YCR	0.022	0.013	0.034	5.9%

*YCR *young children’s resilience, *GI* grandparents’ involvement, *MPS *mother’s parenting stress, *FS *family strength

## Discussion

This study investigated the relationship between grandparents’ involvement and young children’s resilience and elucidate the underlying processes. Drawing upon ecosystem theory, this study investigated the association between grandparents’ involvement and young children’s resilience by developing a chain mediation model to reveal a significant association between the two. Moreover, grandparents’ involvement influenced young children’s resilience partially through the independent mediating roles of maternal parenting stress and family strength, as well as the chain mediating role of maternal parenting stress and family strength. These findings not only imply that grandparents’ involvement in parenting positively impacts young children’s resilience, but also elucidate the internal processes of their interaction, contributing to a theoretical understanding and practical implications.

### The role of grandparents’ involvement in promoting young children’s resilience

Drawing on family systems theory (Minuchin, 1985), this study examined the interplay among child, grandparents, parents, and family variables, demonstrated that grandparents’ involvement could directly or indirectly enhance young children’s resilience by alleviating mother’s parenting stress and strengthening family strength.

Research findings indicate that grandparents’ involvement is positively related to young children’s resilience. In grandparent-co-parenting models, young children also establish stable emotional bonds with their grandparents, forming grandparent-grandchild attachment (Poehlmann, 2003). They develop a sense of belonging to their grandparents’ family and community, exhibit advanced cognitive skills, and demonstrate positive self-worth and family cohesion (Egeland et al., 1993). Furthermore, from a social learning perspective (Bandura, 1969), children acquire self-regulation and initiative by observing grandparents’ behaviors, adopting similar actions in daily life. These findings align with studies affirming the benefits of grandparents’ involvement (Rahmawati & Diana, 2016; Chen & Jiang, 2019), which indicate that grandparents enhance young children’s resilience through positive interactions.

Compared to parents, grandparents, with their extensive life experience, provide greater warmth, care, and nurturing (Song et al., 2016), fostering stable grandparent-grandchild attachment that enhances young children’s emotional regulation (Hayslip, 2019; Wen et al., 2021), improve social adaptation, and reduce behavioral problems (Luo et al., 2020). These findings align with research in Western contexts on the influence of grandparents on young children’s resilience in families affected by single parenthood, divorce, stepfamily dynamics, parental substance abuse, or violence (Downie et al., 2010; Minkler & Fuller-Thomson, 1999). This finding supplied empirical results for the theory of “Cha Xu Ge Ju” (Ma, 2007), which positions young children at the center of concentric interpersonal relationships in Chinese three-generation families, with grandparents playing an indispensable role through caregiving and attention. Grandparents’ involvement not only promotes young children’s resilience and compensates for parental absence in vulnerable families but also strengthens family strength. Moreover, grandparents transmit familial cultural values, fostering grandchildren’s cultural identity and family unity (McAdoo & McWright, 1995; Wiscott & Kopera-Frye, 2000). By responding to family members’ needs for stable, continuous, and communicative relationships, grandparents actively guide grandchildren in understanding contemporary family dynamics, thereby enhancing family identity and strength (Bates & Taylor, 2013a, b).

### The mediating role of mother’s parenting stress and family strength

Previous studies have identified the impact of mother’s parenting stress and family strength on young children’s resilience (Lee & Moon, 2011), as well as mother’s parenting stress as mediator in the relationship between grandparents’ involvement and young children’s outcomes (Luo et al., 2020). However, few studies have delved into the mediating process between mother’s parenting stress and young children’s resilience, although the former can disrupt family systems and adversely affect children’s development.

In today’s rapidly changing society, mothers who are predominantly the primary caregivers and often independently juggle childcare responsibilities can encounter conflicts between work and early childhood parenting, leading to a myriad of stressors. This not only impacts the mothers themselves, but also undermines family cohesion, subsequently influencing young children’s emotional and psychological well-being. Higher stress related to child characteristics among mothers contributes to lower family strength (Park & Moon, 2008). Maternal social support influences maternal parenting stress and behavioral indicators, consequently affecting family functioning and child development (Crnic & Greenberg, 1990). Grandparents’ involvement provides significant social support for mothers by sharing a substantial portion of the parenting burden, thus positively mitigating maternal parenting stress (Noriega et al., 2017).

Psychological factors within the family environment are crucial variables for predicting children’s future positive adjustment (Owens & Shaw, 2003). The closeness and rapport among family members demonstrate warm, cooperative, and supportive patterns of interaction, fostering strong bonds between young children and their family members (Mchale, 2007). In families characterized by high family strength, encountering a crisis or problem fosters democratic communication among family members. This fosters strong familial bonds, shared values, and effective crisis resolution through dialog. Viewing family crises as opportunities for growth positively impacts the development of increased resilience in young children (Lee et al., 2009). This strong sense of bonding and emotional closeness among family members, along with a successful family environment, positively impact family relationships as well as the development of resilience in young children (Kim et al., 2007).

Additionally, the results revealed a process model of “grandparents’ involvement-mothe’s parenting stress-family strength-young children’s resilience”. Specifically, grandparents’ involvement not only indirectly promotes young children’s resilience by reducing mother’s parenting stress and enhancing family strength, but it can also be seen that grandparents’ involvement promotes young children’s resilience through a dual mediation effect. These preliminary findings align with research on grandparents across diverse cultural and family contexts (Luo et al., 2020; Choi, 2006; Jo & Noh, 2015). As “stress-buffers” within the family (Botcheva & Feldman, 2004), grandparents can provide effective advice and assistance to mothers of young children facing stress (Li et al., 2016). Grandparents’ involvement in childcare not only moderates maternal parenting stress but also helps transmit family culture, enhance intimacy among family members, and strengthen family cohesion (Sun & Mulvaney, 2021). Mothers with lower levels of parenting stress are better able to adopt appropriate parenting behaviors to respond to young children’s needs, thereby promoting the development of children’s social adaptation skills and resilience (Kim, 2005). This effect is particularly pronounced in Chinese three-generation families, where interdependent and closely interactive family dynamics prevail. Such families with grandparental involvement can cultivate positive symbiotic relationships (Luo et al., 2020).

## Implications, limitations, and conclusions

### Practical implications

First, the influence of grandparents’ support on young children and families warrants careful consideration in the ongoing enhancement of future childcare welfare systems and retirement pension schemes. Grandparents’ engagement in early childhood parenting serves as a valuable resource by aiding mothers in cultivating positive family dynamics amid adversity, navigating the challenges of motherhood, and enhancing the family’s cohesion. However, relying solely on grandparents to address familistic caregiving is unsustainable, child-rearing responsibilities should be shared among families, society, and the state. To support low-fertility and aging societies, families, society, and the state must collaboratively foster a caregiving culture and establish compensatory mechanisms for child-rearing.

Second, in the realm of social education, mothers should receive guidance on not only parenting and fostering mental resilience in young children, but also nurturing improved interactions with grandparents to facilitate their constructive involvement in early childhood caregiving. In addition, care must be taken to prevent the caregiving demands of grandchildren from translating into stress for grandparents, with families and society prioritizing the physical and psychological well-being of grandparents.

Moreover, educational initiatives highlighting intrinsically healthy family dynamics can promote active family engagement in resolving issues through fostering constructive dialogs, either through counseling, intervention, or educational platforms. In such nurturing environments, young children can experience both physical and psychological security.

Finally, grandparents’ active participation in early childhood caregiving fosters heightened interactions with their grandchildren, leading to affection from their grandchildren. Consequently, enhancing positive sentiments toward grandparents and the elderly during early childhood is a foundational approach in tackling both early childhood parenting and elder-related concerns. Furthermore, grandparents’ involvement in parenting behaviors has multifaceted effects on young children and holds implications for the future of society, warranting its promotion.

### Limitations and implications for future research

Future research should account for pronounced cultural differences in gender roles and the historical caregiving roles of women, examining the influence of grandmothers (women) and grandfathers (men) on childcare.

Secondly, beyond exploring the relationships between grandparents and young children, as well as grandparents and parents, studies should incorporate the effects of these intergenerational dynamics on young children’s resilience. Furthermore, integrating diverse social-environmental variables associated with young children’s resilience will enable more comprehensive and ecologically informed investigations.

Third, although this study established the impacts of grandparents’ involvement on young children’s resilience and its pathways of action, it inherently remains a correlational study, precluding definitive causal inferences. Subsequent research endeavors should validate the causal relationship between grandparents’ involvement and young children’s resilience through longitudinal or experimental studies.

Fourth, strategies should be developed to address the limitations of maternal self-report questionnaires. For instance, grandparents’ self-assessments of their caregiving involvement or fathers’ observations of grandparental participation could provide more objective insights. Similarly, young children’s resilience could be evaluated through objective measures, such as teacher assessments, high-quality observational methods, or interviews.

Finally, future research should conduct studies across diverse regions to overcome the geographical limitations of current samples.

## Conclusions

This study ultimately highlights the following two points. First, grandparents’ involvement positively predicts resilience in young children. Second, grandparents’ involvement influences young children’s resilience partly through independent mediating effects, including the mother’s parenting stress and family strength, as well as the chain mediating effect of mother’s parenting stress and family strength.

## Data Availability

The data that support the findings of this study are available from the corresponding author, Leishan Shi, upon reasonable request.
